# Terrestrial Snakebites in the South East of the Arabian Peninsula: Patient Characteristics, Clinical Presentations, and Management

**DOI:** 10.1371/journal.pone.0024637

**Published:** 2011-09-12

**Authors:** Juma M. Alkaabi, Mariam Al Neyadi, Fakhra Al Darei, Mariam Al Mazrooei, Jawaher Al Yazedi, Abdishakur M. Abdulle

**Affiliations:** 1 Department of Internal Medicine, Faculty of Medicine and Health Sciences, United Arab Emirates University, Al Ain, United Arab Emirates; 2 Department of Internal Medicine, Buraimi Hospital, Buraimi, Sultanate of Oman; University of Pittsburgh Medical Center, United States of America

## Abstract

**Background:**

To describe the characteristics, clinical presentations, management and complications of snakebites in the border region between Al-Ain, United Arab Emirates (UAE) and Buraimi, Sultanate of Oman.

**Methodology/Principal Findings:**

We carried out a retrospective review of medical records to study snakebite cases over four-year duration at three tertiary hospitals. Overall, 64 snakebite cases were studied with median hospitalization of 2 (interquartile range [IQR] 1–4) days. The majority of cases were male (87.5%), and most (61%) of the incidents occurred during summer months. The bite sites were predominantly (95%) to the feet and hands. Main clinical features included pain, local swelling, and coagulopathy, blistering and skin peeling. Overall, there were no deaths, but few major complications occurred; extensive skin peeling (n = 5, 8%), multi-organ failure (n = 1, 1.5%), and compartment syndrome (n = 1, 1.5%). Polyvalent anti snake venom (ASV), analgesia, tetanus toxoid, intravenous fluids, and antibiotics such as ampicillin, cloxacillin, and cephalosporins were commonly instituted as part of treatment protocols in the three hospitals.

**Conclusion:**

The overwhelming majority of bites occurred during summer months, and envenomations were more common in, relatively, young male farmers, but with no serious clinical complications. Prevention and treatment strategies should include increasing public awareness, developing management guidelines, and manufacturing specific ASV for a wide spectrum of the local venomous snakes.

## Introduction

Snakebites are a common problem in many parts of the world and account for a considerable morbidity and mortality due to envenomation [1], [2]. Recent studies have shown that snake envenomation is more common among poor communities with the highest burden of mortality being reported in countries least able to cope with the high cost of anti snake venom (ASV) treatment [3]. Such burden has been reported in, among other countries, South and Southeast-Asia, and Sub-Saharan Africa [2], [4]. However, little is known about the magnitude of snakebites in the Middle East, and more so in the Arabian Gulf countries.

Whilst, snakebites are not widely reported in this part of the world, available data in the literature indicates that the most common venomous snakes in the Middle East are vipers; *Cerastes gasparetti*, *Echis carinatus*, *Echis coloratus*, and *Pseudocerastes persicus* [5], [6], [7], and non-vipers; mainly C*olubridae*, *Atractaspididae* and *Elapidae species* [8], [9].

**Figure 1:**
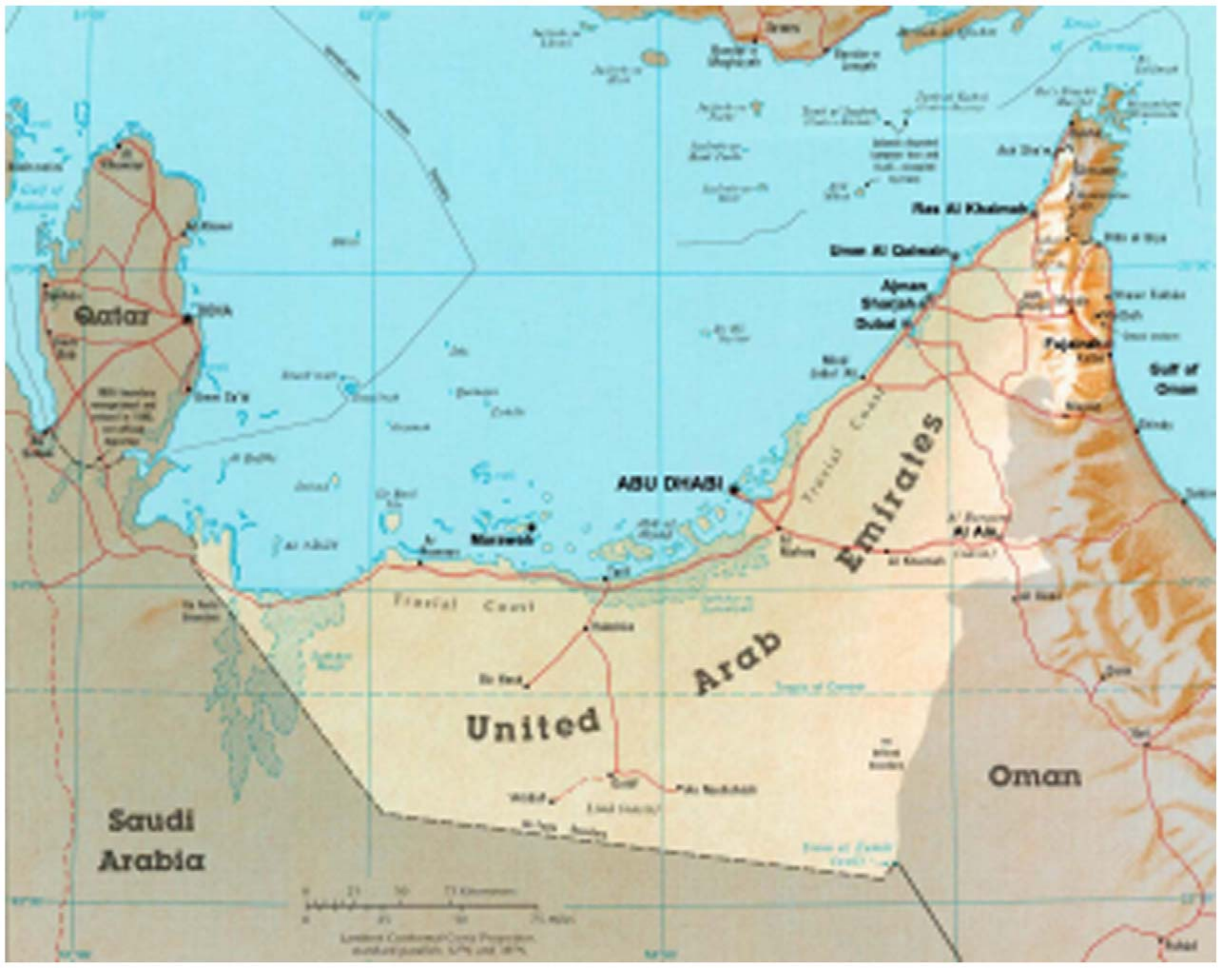
Map illustrating the location of Al-Ain, United Arab Emirates, and Buraimi, Sultanate of Oman.

The in-land and arid ecoregion in the South Eastern part of the Arabian Gulf i.e. United Arab Emirates (UAE) and Oman comprises sparse vegetations, sand dunes, gravel plains, rugged mountains, and scattered oases. In particular, the climate in Al-Ain and Buraimi cities, located in the border between the two countries (Figure 1), is relatively less humid, though warm in nature, with abundant agricultural activities as compared to the major coastline cities of the two countries. Such climate may constitute a favorable habitat for higher incidents of snakebites [2]. We, therefore, carried out a retrospective case series study to review medical records with the aim of describing patient characteristics, clinical presentations, and management of snakebites in three regional tertiary hospitals.

## Materials and Methods

This retrospective case series study was conducted by the Department of Internal Medicine, Faculty of Medicine & Health sciences, UAE University in collaboration with three main hospitals in the region; Tawam, and Al-Ain hospitals; Al-Ain, UAE, and Buraimi Hospital; Buraimi, Sultanate of Oman. The three hospitals provide emergency services in the region around the clock. The estimated catchment population size in the region is approximately 650,000 (Al Ain 500,000 and Buraimi 150,000). Participating hospitals were selected based on the realization that ASV was available only at these government hospitals. Medical records were reviewed if the final discharge diagnosis indicated an incident of snakebite based on the electronic registry of the study sites (hospitals). Snakebite was recorded if the subjects have seen the snake or if the appearance of the puncture sites was convincingly of snakebite. The exclusion criteria included any other forms of bites which are not in keeping with snakebites. Cases of snakebites were reviewed over duration of four years from December 2005 to December 2009. A data collection sheet was used to extract relevant information pertaining patient characteristics, clinical presentations, laboratory investigations, and treatment regimens. Three medical students collected the data as part of a summer research assignment and the students were involved in the study design, data collection, and data interpretation process. Prior to data collection, students were given orientation sessions on how to extract uniform and accurate data from the medical records. To minimize interpersonal variability in the data collection process, all students worked as a team. The collection sheet was pre tested in 10 cases and, where applicable, changes were made accordingly. Subsequently, the principal investigator reviewed the quality and accuracy of the collected data before and after data entry. Given the retrospective nature of the study, there was no direct involvement of human subjects, and thus no written or verbal consent was necessary. The protocols of this study were approved by the Al-Ain Medical District Human Research Ethics Committee, and Buraimi Hospital.

### Statistical Methods

Standard descriptive and analytical statistical methods for univariate and bivariate analysis, such as mean, median, standard deviation (SD), Chi-square, Mann-Whitney tests and Spearman's correlation coefficient were used. For all analyses SPSS v18.0 was used. P-values<0.05 (2-sided where applicable) were considered statistically significant.

## Results

**Table 1** shows the demographic characteristics of the study population. A total of sixty-four cases (87% males) of snakebites were studied. On average, the annual frequency of snakebite was 6 in Buraimi; 6 in Al Ain; and 4 in Tawam. There were no statistically significant changes in the number of snakebite cases between the four studied years (P<0.6). This might, however, be attributed to the short duration of the observation and the small number of cases per year per facility.

**Table 1:** Demographic characteristics of the studied population (n = 64).

Parameter	Sub-groups	Number (%)
Age (mean ± SD)		30.9±14.06
Gender	Male	56 (87.5)
	Female	8 (12.5)
Hospitals	Al-Ain	25 (39.1)
	Tawam	16 (25)
	Buraimi	23 (35.9)
Season of the bites	Winter	6 (9.4)
	Spring	8 (12.5)
	Summer	39 (60.9)
	Autumn	10 (15.6)
Time of the bite	Morning	20 (31.3)
	Afternoon	16 (25)
	Evening	19 (29.7)
	Night	7 (10.9)
Site of the bite	Feet	37 (57.8)
	Hands/arms	24 (37.5)
	Others	3 (4.7)
	Time to reach ER (mean ± SD; range) hrs	2.9±3.5; 1–18
	Hospitalizations: median (IQR) days	2.00 (1–4)
	Previous snakebite	5 (8)

Demographic characteristics of the study sample (n = 64): Shows the characteristics of the study subjects; numbers, and percentage and where applicable mean ± standard deviation, range, median and interquartile range (IQR). ER; emergency room, SD; standard deviation, IQR; interquartile range.

Patients' mean age ± SD (standard deviation) was 30.9±14.0 years. Most of the bites (60%) occurred during the summer months of June, July, and August and in the morning and afternoon. The most frequent sites of bites were to the feet (57%) and the hands or arms (37%). The majority (60%) of snakebite incidents were reported among male farmers often during farming activities. The mean interval time to reach emergency room (ER) was 2.9 hours ranging from 1–18 hours. The median hospitalization duration was 2 (IQR:1–4) days. Only five subjects (8%) reported previous history of snakebites. No definite types of the involved venomous snakes were identified despite reporting that “snake was brought dead” in some of the medical records.

Clinical symptoms and signs, laboratory findings, and management regimens are summarized in **Table 2**. The common features of clinical presentation were pain, swelling, bleeding or oozing of fluid from the wound site. Localized oedema, blisters or skin peeling developed within few days of admission. Though 27% of patients reported muscular weakness, neurological examinations were unremarkable. There were no reported impaired consciousnesses, hypotension, shock or rhabdomyolosis. All patients were fully conscious, haemodynamically stable and had normal oxygen saturation at room air. Derangements of the coagulation profile were found in 36 (56%) of the patients. There was one case with thrombocytopenia (28×10^9^/L). All laboratory abnormalities were transient and normalized after 2–4 days of treatment. The median hospitalization duration was 2 (IQR:1–4) days. One elderly diabetic patient was hospitalized for 76 days due to multi-organ system failure. This patient had a complex clinical history involving prolonged hospitalization with multiple organ failure including coagulopathy, hepatic impairment, renal, respiratory failure and multiple systemic infections. Three patients (4.7%) were readmitted with coagulopathy following initial normalization of their coagulation profile prior to discharge. The majority of patients had a benign course with complete recovery. Four patients (6%) had bleeding gums. One patient (1.5%) underwent hand fasciotomy following the development of compartment syndrome (tight tourniquet was applied prior to admission).

**Table 2:** Clinical presentations and laboratory findings among the study subjects.

Parameter		Number (%)
Symptoms		
	Local pain	57 (89.1)
	Local swelling	47 (73.4)
	Local parasthesia	11 (17)
	Local bleeding	8 (12.5)
	Muscular weakness	17 (27)
	Gum bleeding	4 (6)
Signs	Local redness	31 (48)
	Local oedema	44 (69)
	Local blisters	4 (6)
	Fever	4 (6)
	Cellulitis	14 (22)
	Skin peeling	5 (8)
	Wound oozing	9 (14)
Laboratory findings (mean ± SD)		
INR (normal range = 0.8–1.2)	On admission	1.47±1.30
	Highest as in-patient	2.82±3.03
	On discharge	1.09±0.11

Clinical features and presentation among the snakebite patients. The table displays the symptoms, signs, and laboratory findings expressed in percentage among the study subjects. INR; International Normalized Ratio.

With regard to laboratory findings, there was no statistically significant association (Mann-Whitney test) between hospitalization time as an outcome and the site of the bite, gender, age, early arrival to the ER, initial levels of the International Normalized Ratio (INR) or Partial Prothrombin Time (PT) (Spearman's correlation), as well as administration of ASV, number of ASV vials received or the use of antibiotics. There was no association between presence of local complications and longer hospitalization duration. On follow up, fourteen patients (22%) reported persistent localized mild pain or paraesthesia at the bite site and there were no other reported major complications or disabilities (≥3 months post-discharge).

All patients (100%) were given simple analgesia of paracetamol and/or NSAIDs (Non-Steroidal Anti-Inflammatory Drugs); whereas only 62%, 75%, and 60% of the patients were given ASV, tetanus toxoid (single injection), and antibiotics respectively.

**Figure 2:**
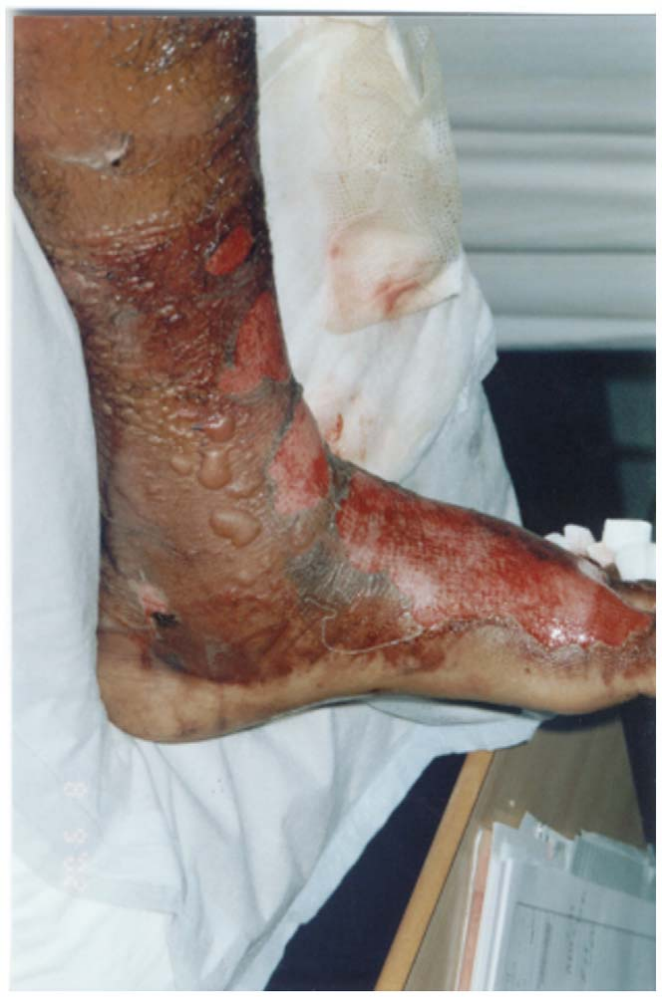
Snakebite to the dorsum of the left foot resulting in swelling, bullae formation and skin peeling.

The ASV used in the three hospitals was equine polyvalent ASV (The Antivenom &Vaccine Production Center, National Guard Health Affairs, Riyadh, Saudi Arabia). Each vial contained 10 ml of purified immunoglobin fractions against five types of snakes (*Bitis arietans*, *Cerastes cerastes*, *Echnis carinatus*, *Echis coloratus*, *and Naja haje*). Of the studied population, forty patients (62.5%) received ASV treatment. Noticeably, there was significant variations between the three hospitals in the practice of ASV administration; Al-Ain (48%), Tawam (50%) and Buraimi (87%). The mean dose of the administered ASV was (4.9±5.8; range 0–25 vials), with no documented major side effects. However, anti-histamines were given to 12 patients (19%) and two patients (3%) received short course of steroids (indications were not clearly defined). No epinephrine use was found. Moreover, 48 patients (75%) were given tetanus toxoid. All studied subjects received intravenous fluids and simple analgesia. Antibiotics such as ampicillin, cloxacillin and cephalosporins were commonly administered to treat local open wounds, and tissue necrosis. In our series, thirty-nine patients (70%) received antibiotics. Again, significant variations existed in the administration of antibiotics in the three hospitals; Al-Ain (28%), Tawam (75%) and Buraimi (87%). Figure 2 shows an example of extensive skin peeling and bullae formation in one of the studied cases.

## Discussion

In this study, we documented several important findings in relation to patient characteristics, clinical presentations, and current management of snakebites in the border region between UAE and Sultanate of Oman.

The overwhelming majority (87%) of the studied cases were relatively young male farmers. This is most likely due to occupational exposure, as males are more involved in farming activities as opposed to females. Our findings are therefore in keeping with available data in the literature [6], [10]. All female subjects, but one, were seen at Buraimi hospital. The reasons behind such isolated high incidence of snakebites among females in Buraimi is not very clear, especially that Buraimi has far less population than Al-Ain. The existence of more scattered small farms in the mountainous habitats of Buraimi as opposed to Al-Ain, may explain, in part, such isolated and high incidents of snakebites among females subjects in Buraimi. Most of the snakebite incidents occurred in the summer months especially during the morning, afternoon, and early evening as opposed to late night exposure. Understandably, snakes like all reptiles are ectotherms relying on the sun for warmth hence faster mobility during daytime. The most common bite sites were the feet, suggestive of the lack of protective footwear among farmers. It is worth noting that wearing slippers/sandals is rather common in this part of the world even while farming. Clearly, these findings raise important questions with regard to the need to re-enforce adequate safety measures that could minimize occupational hazards among farm workers in the region. We did not collect data regarding the exact circumstances of snakebites, thus, we could not comment on the specific occupational nature of snakebite incidents.

No fatalities were reported and most, if not all, of the subjects were taken to hospital within a reasonable timeframe. The mean interval time to reach the nearest emergency unit (2.9 hr) is indeed far better than what has been reported elsewhere (1–72 hr) in the region [7]. It is not clear, however, whether this is due to hospital proximity or an increased population awareness regarding the deleterious consequences of snakebites. Nonetheless, our data does not rule out the possibility that some of the snakebite victims may have been taken to other hospitals or even died prior to reaching hospital facilities (personal communication; death of a young man in a camp due to multiple snakebites). Hence, our findings may underestimate the actual burden of snakebites. The modest duration of hospitalization reported here, does indicate that most of the cases may have been presented with mild to moderate clinical complications. Although our data is limited and no snakes were identified by species, the observed clinical presentations and complications may suggest vipers, commonly found in the Middle East, as the most likely culprits [7], [11], [12].

The clinical presentations (symptoms, signs, and laboratory findings) were in general of mild in nature. Local swelling, inflammation, and coagulopathy were common manifestations of snakebites. No other forms of systemic envenomation were documented such as hypotension, shock, rhabdomyolysis, neurotoxicity or impaired consciousness. Of the total subjects studied, only two cases (3%) reported to have used tourniquets, one of which has developed compartment syndrome of the right hand necessitating faciotomy. The value of pressure immobilization method is controversial [13]. Some venom, such as that of Cobra and Viper, primarily produce local necrosis and thus localization of toxins may worsen the syndrome. Other potential limitations with pressure immobilization method include lack of easily available and suitable material as well as the difficulties associated with determining the necessary pressure to apply i.e. if the compression is too loose, it will fail to work; if too tight, it may obstruct arterial flow thus leading to local ischemia and necrosis. Although, at least one patient had incision marks at the bite site carried out by a family member, no traditional first-aid manipulations i.e. incision, suction, use of caustics, oxidizing agents or cryotherapy were reported.

These findings suggest that the sequelae of snakebites in the studied population are of mild in nature as compared to the situation in other parts of the world, particularly, Africa, Middle East and Indian Subcontinent. This is, presumably, because of less venomous species found in this region [14].

With regard to the management of snakebites, localized pain was often controlled with simple analgesia with the exception of few cases were pethidine was used instead. It is worth noting that pethidine or morphine administration should be used with caution to avoid potentiating respiratory depression in patients with certain snakebites. In the current local practice, there is only one available polyvalent ASV and thus identification of the type of the snake by the treating physicians will not change the protocol of treatment. A large proportion of our series (62%) were given ASV but premedication with epinephrine or steroids prior to the ASV were not given. Two patients received steroids at later stage of the management (indications were not clear). The main indications for the use of ASV were due to significant local swelling or local tissue necrosis. Comparing the three hospitals there was clear variation in the clinical indications of starting ASV or antibiotics.

We observed excessive use of ASV treatment by physicians. Such practice, though may not be warranted, may have occurred due to uncertainty about the type of snake, the nature of the wound, and indeed absence of local guidelines.

ASV administration is generally not recommended for patients with minimal swelling and no signs of systemic envenomation. Fortunately, no anaphylactic reactions or serum sicknesses were recorded in our study. Only 75% of the patients in this retrospective study received tetanus toxoid. Previously, some cases of tetanus were reported [15], leading to the isolation of *Clostridium tetani* from snake's oral flora [16]. As a result, the administration of a booster dose of tetanus toxoid has been recommended following snakebites [17]. Approximately, 60.9% of our studied population received antibiotic treatment, compared to 89% in a similar study in Saudi Arabia [7] of which antibiotics were given mainly due to suspected or proven wound infection.

Often times, signs of local inflammatory reaction can be easily misdiagnosed as microbial infection. Snake venom causes tissue destruction and may expose the wound to bacterial infection [18]. However, the use of prophylactic antibiotics is somewhat debatable [19]. Among other factors, the spectrum of bacteria from the venom and oral cavities of snakes varies from not only different species, but also from different geographical regions [20]. In our study, there were clear variations in the use of antibiotics as well as the type of antibiotics used in the different hospitals. Whilst the overwhelming majority of patients treated in Tawam Hospital and more so in Buraimi Hospital were given prophylactic antibiotics, the practice was far less common in Al-Ain Hospitals. Nonetheless, the majority of the antibiotic use was empirical and without reported culture and sensitivity. Such wide-spread use of antibiotics is of concern, and thus there is a significant opportunity to improve the current practice in the management of snakebite wounds.

Together, the above shortcomings suggest a pressing need to establish national registry and indeed treatment guidelines for the management of snakebites in this part of the world. Such registry, we think, may help not only to collect accurate and detailed data about snakebites, but also obtaining data comparable with the available literature. Moreover, manufacturing specific ASV for the local terrestrial snakes is expected to yield better results.

### Limitations

Our study has some limitations. First, the retrospective nature of the study may have limited our ability to collect precise and detailed information. Consequently, we could neither classify the severity of the symptoms and signs, nor assess the impact of treatment based on the current findings. In addition, identifying adverse reactions to treatment modalities were difficult due to lack of accurate documentation. Second, possibility is high that a number of patients did not seek medical treatment in the three main study hospitals where the study was conducted, thus leading to a possible underestimation of the actual incidents of snakebites in the region. Third, our study subjects were from the border towns between UAE and Oman, therefore, the results cannot be generalized to the wider population of these two countries.

### Conclusion

We conclude that the overwhelming majority of snakebites occurred mainly among young male farmers, often during summer months, but with no serious clinical complications. There is significant variability in the management of snakebites in the three study hospitals. Adequate prevention and treatment strategies could include identifying local snakes, developing management guidelines, and increasing public awareness. Moreover, manufacturing specific ASV targeting the spectrum of the local venomous snakes might be advantageous.
